# Bruxism in children and transverse plane of occlusion: Is there a relationship or not?

**DOI:** 10.1590/2176-9451.19.5.067-073.oar

**Published:** 2014

**Authors:** Ana Carla Raphaelli Nahás-Scocate, Fernando Vusberg Coelho, Viviane Chaves de Almeida

**Affiliations:** 1 Associate professor, Undergraduate and Postgraduate Program in Orthodontics, University of São Paulo City - UNICID; 2 DDS, UNICID; 3 MSc in Orthodontics, UNICID

**Keywords:** Malocclusion, Bruxism, Epidemiology

## Abstract

**OBJECTIVE:**

To assess the occurrence of bruxism in deciduous dentition and a potential association between the habit and the presence or absence of posterior crossbite.

**METHODS:**

A total of 940 patient files were assessed. They were gathered from the archives of University of São Paulo City - UNICID; however, 67 patient files were dismissed for not meeting the inclusion criteria. Therefore, 873 children, males and females, comprised the study sample. They were aged between 2-6 years old and came from six different public primary schools from the east of the city of São Paulo. Data were collected through questionnaires answered by parents/guardians and by clinical examinations carried out in the school environment in order to obtain the occlusal characteristics in the transverse direction. First, a descriptive statistical analysis of all variables was performed (age, sex, race, posterior crossbite, bruxism, headache and restless sleep); then, the samples were tested by means of chi-square test with significance level set at 0.05%. A logistic regression model was applied to identify the presence of bruxism.

**RESULTS:**

The prevalence of this parafunctional habit was of 28.8%, with 84.5% of patients showing no posterior crossbite. Regarding the association of bruxism with crossbite, significant results were not found. Children with restless sleep have 2.1 times more chances of developing bruxism, whereas children with headache have 1.5 more chances.

**CONCLUSION:**

Transverse plane of occlusion was not associated with the habit of bruxism.

## Abstract (pt)

**INTRODUÇÃO:**

avaliar a prevalência de bruxismo na dentadura decídua e a associação existente entre este hábito e a presença ou não de mordida cruzada posterior.

**MÉTODOS:**

foram avaliados 940 prontuários do arquivo de documentações da Universidade Cidade de São Paulo (UNICID), sendo que 67 foram excluídos por não atenderem aos critérios de inclusão. Logo, 873 , de ambos os sexos, na faixa etária de 2 a 6 anos, procedentes de seis escolas municipais de educação infantil da zona leste da cidade de São Paulo, compuseram a amostra desse estudo. Os dados foram coletados por meio de questionários respondidos pelos pais/responsáveis e exames clínicos, realizados em ambiente escolar, para obtenção das características oclusais no sentido transversal. Primeiramente, efetuou-se uma análise estatística descritiva de todas as variáveis avaliadas na amostra (idade, sexo, raça, mordida cruzada posterior, bruxismo, dor de cabeça e sono agitado) e, em seguida, utilizou-se o teste qui-quadrado, com grau de significância de 0,05%, e um modelo de regressão logística para a presença do bruxismo.

**RESULTADOS:**

a prevalência desse hábito parafuncional foi de 28,8% do total da amostra, e 84,5% não apresentaram mordida cruzada posterior. Quanto à associação de bruxismo com a mordida cruzada posterior, não foram encontrados resultados significativos. Verificou-se, também, que as crianças com sono agitado possuem 2,1 vezes mais chances de desenvolver o bruxismo e, as com dor de cabeça, 1,5 vezes.

**CONCLUSÃO:**

o plano transversal de oclusão não apresentou relação com o hábito do bruxismo.

## INTRODUCTION

Bruxism is a parafunctional activity of the masticatory system characterized by clenching or grinding the teeth with rhythmic muscular contractions more frequent during sleep.^28^ While this parafunctional activity is performed, nearly entirely in the subconscious level, neuromuscular protection mechanisms are absent,^19^ thereby changing the masticatory system and causing temporomandibular disorders.^29^

Clinically, bruxism in children not only causes different levels of tooth surface wear, but also provides patients with muscle and joint discomfort. Furthermore, due to axial forces generated in patient's teeth, it can act as an adjuvant in the progression of destructive periodontal disease in children,^2^ in addition to contributing to the development of false Class III, accelerated root resorption of deciduous teeth, changes in the chronology of permanent teeth irruption and promotion of dental crowding.^15^ Thus, this habit should be diagnosed and managed as early as possible.^15^

There have been only a few published studies conducted to assess the prevalence of bruxism in children. This limitation prevents scientific parameters and association of bruxism with etiological factors to be established. The literature appears to be contradictory regarding the etiology of this parafunctional habit in childhood, thereby characterizing its origin as multifactorial, involving heredity, psychological and behavioral factors as well as occlusal interferences and certain pathologies.^4^^,^ 14 ^,^ 17 ^,^ 18 ^,^ 20 ^,^ 21 ^,^ 28 Some authors report that occlusal factors such as overjet, overbite, molar and canine relationship, open bite and crossbite might play an important role in the development of this habit in children.^12^^,^ 26 ^,^ 29

Bruxism in children is also diagnosed with painful symptoms of Temporomandibular Disorders (TMD) of which it can be considered a possible causal factor.^26^ Additionally, frequent headaches and restless sleep are associated with this habit.^3^

Thus, in this context, this study aimed at investigating the prevalence of bruxism in deciduous dentition, trying to relate it to occlusal factors, specifically the transverse plane of occlusion, so as to establish a causal factor alone, since several nuances are present in scientific research. In addition, the association of this parafunctional habit with restless sleep and headaches was also verified.

## MATERIAL AND METHODS

### Ethical aspects

This study was funded by the Brazilian National Council for Scientific and Technological Development - CNPq (Undergraduate Research) from 2008 to 2009. It is part of a project developed to assess the association between bruxism in children and posterior crossbite, conducted in accordance with the rules and principles adopted by Ethics and Research (13355774).

### Sample selection

Clinical records of Brazilian children, males and females, aged between 2 years and 1 month old and 6 years and 11 months old, enrolled in 2005 in primary public schools located in the east of the city of São Paulo, were assessed. The initial sample comprised 940 children; however, 67 records were excluded for not meeting the inclusion criteria. Therefore, the final sample comprised 873 children of which 37.3% and 38.7% were 4 and 5 years old, respectively.

### Inclusion criteria

The medical records of children included in this sample met the following inclusion criteria: Informed consent form signed by parents/guardians; questionnaire properly answered; children aged between 2 years and 1 month and 6 years and 11 months; complete deciduous dentition without erupted or erupting permanent teeth; absence of carious lesions or extensive loss of structure that compromised coronary occlusion; lack of early loss of deciduous teeth; absence of any kind of trauma; absence of visual and/or hearing and/or mental impairment; no previous orthodontic treatment and/or speech therapy.

### Informed consent form and questionnaire

Parents/guardians were given detailed explanation about the objectives and design of data collection in the schools. Subsequently, an informed consent form was sent so as to notify them about the research, its importance and objectives. A questionnaire on parafunctional habits and other characteristics related to the overall health of children was also sent. The examiner did not interfere during questionnaire completion.

### Examiner calibration

Three examiners were properly calibrated prior to clinical examination. The calibration process included the examination of occlusion in 24 children, performed twice with a 15-day interval in between. Data were subjected to Kappa statistics (K) for analysis of reproducibility. An index greater than 0.81 K and Pearson correlation coefficient (Rs > 0.90) were calculated. Results revealed excellent intra and inter-examiner reliability.

### Clinical examination

All clinical examinations were performed in the school environment with the children sitting comfortably and facing abundant source of artificial light. First, they were asked to perform maximum mouth opening and then occlude in maximum intercuspation (MIH) for data collection.

To assess transverse posterior relationship between upper and lower dental arches, the following criteria were applied: Absent posterior crossbite (when the palatal cusps of upper posterior teeth occluded in the groove of mandibular posterior teeth), and presence of posterior crossbite (when the buccal cusps of upper posterior teeth occluded in the central sulcus of lower teeth, thereby establishing a transverse or inverted cross relationship on the back). Subsequently, both unilateral crossbites, whether with shift or true, as well as bilateral ones, were included.

### Questionnaire

A questionnaire^13^ comprising standardized questions about patient's history was answered by parents/guardians.^17^ Based on the questionnaire, the presence of bruxism (yes or no), the period in which the child presented the parafunctional habit (daytime, night time or both), and the variables related to the presence of restless sleep (yes or no) as well as headaches (yes or no) were investigated. It is worth mentioning that the examiners did not interfere while the questionnaires were being filled out.

### Data analysis

First, a descriptive statistical analysis of all variables (age, sex, race, posterior crossbite, bruxism, headache and restless sleep) was carried out. Subsequently, analyses of statistical significance were conducted by means of chi-square test in order to investigate possible associations between bruxism and other characteristics (sex, race, posterior crossbite, headache and restless sleep). The same test investigated a potential association between posterior crossbite, headache and restless sleep. At last, a logistic regression model was applied for the presence of bruxism, thus presenting the Odds Ratio (OR). The level of significance was set at 5%, i.e., test results were not statistically significant when P-value was less than or equal to 0.05.

## RESULTS

### Descriptive analysis of variables in the sample

Sample distribution according to age was predominantly composed of children between 4 and 5 years old (37.3% and 38.7%, respectively). Regarding patients' sex, the sample was very well divided and comprised 434 females (49.7%) and 439 males (50.3%). As for race, most children had brown skin (pardo) (58%), whereas only 0.4% had yellow skin.

The minority showed posterior crossbite, totaling 15.5% (n = 135) as distributed in Table 1. As for the prevalence of the parafunctional habit of bruxism, 26.5% of children had the habit at night, 2.2% during the day and 0.1% during the night and during the day, thereby representing a total of 28.8% of children with bruxism. Out of the total sample, 21.8% (n = 190) reported having headaches while 36.1% (n = 315) claimed to have restless sleep.

**Table 1:** Sample distribution according to posterior crossbite (PCB)

PCB	Absent	Bilateral	Unil. shift R	Unil. shift L	Unil. true. R	Unil. true. L	Total
n	738	19	53	28	21	14	873
%	84.5	2.2	6.1	3.2	2.4	1.6	100

### Association between bruxism and other variables

To assess a potential association between the presence of bruxism and other variables, the sample was divided as follows: "No" (when the child did not have bruxism) and "Yes" (when children showed signs of bruxism at night and/or day). The variable posterior crossbite was also treated in a similar way: "Absent" (when the child did not have posterior crossbite) and "Present" (when the child presented any type of posterior crossbite).

According to Table 2, the prevalence of bruxism was higher for males (30.5%) in comparison to females (27%). Likewise, black-skin children had higher prevalence (36.1%) when compared to other races. Among children with no posterior crossbite, the percentage of bruxism (30.9%) was higher than children with posterior crossbite (17%). As for headaches, 38.4% reported headache and showed signs of bruxism, whereas 26.1% reported having no headache even though they showed signs of bruxism. Children with restless sleep presented a higher percentage of bruxism (39.7%) in comparison to those who did not report restless sleep (22.6%).

**Table 2:** Sample distribution according to the presence of bruxism and variables of sex, posterior crossbite, headache, restless sleep and race.

Bruxism	Sex				Posterior crossbite				Headache				Restless sleep				Race							
	Female		Male		Absent		Present		No		Yes		No		Yes		Yellow		White		Black		Brown	
	n	%	n	%	n	%	n	%	n	%	n	%	n	%	n	%	n	%	n	%	n	%	n	%
No	317	73	305	69.5	510	69.1	112	83	505	73,9	117	61.6	432	77.4	190	60.3	4	100	225	74.5	39	63.9	354	70
Yes	117	27.0	134	30.5	228	30.9	23	17	178	26.1	73	38.4	126	22.6	125	39.7	0	0	77	25.5	22	36.1	152	30
Total	434	100	439	100	738	100	135	100	683	100	190	100	558	100	315	100	4	100	302	100	61	100	506	100

Table 3 presents the results of the chi-square test with significance level set at 5%. Results show a significant association between posterior crossbite (p = 0.0015), headache (p = 0.0012) and restless sleep (p < 0.0001).

**Table 3:** Chi-square test results. Chi-square

Chi-square test	
Comparison with bruxism	P-value
Sex	0.2762
Race	0.1592
Posterior crossbite	0.0015*
Headache	0.0012*
Restless sleep	‹ 0.0001*

* Significant at 5%.

### Association between posterior crossbite, headache and restless sleep

Table 4 shows the result of sample distribution according to the presence of posterior crossbite, headache and restless sleep. Table 5 shows the result of sample distribution according to the presence of headache and restless sleep. A total of 31.7% of children who had restless sleep also had headaches. This represents nearly the double if compared to children who did not have restless sleep (16.1%). The chi-square test showed that there was only significant association between headache and restless sleep with p < 0.0001 (Table 6).

**Table 4:** Sample distribution according to the presence of posterior crossbite associated with headache and restless sleep.

Posterior crossbite	Headache				Restless sleep			
	No		Yes		No		Yes	
	n	%	n	%	n	%	n	%
Absent	579	84,8	159	83,7	471	84,4	267	84,8
Present	104	15,2	31	16,3	87	15,6	48	15,2
Total	683	100	190	100	558	100	315	100

**Table 5:** Sample distribution according to the presence of headache and restless sleep.

Headache	Restless sleep			
	Absent		Present	
	n	%	n	%
Absent	468	83.9	215	68.3
Present	90	16.1	100	31.7
Total	558	100	315	100

**Table 6:** Chi-square test results.

Chi-square test	
Comparison	P-value
PCB X headache	0.7997
PCB X restless sleep	0.9671
Headache X restless sleep	‹ 0.0001*

* Significant at 5%.

### Logistic regression model for the presence of bruxism

A logistic regression model was applied for the presence or absence of bruxism (Table 7). Children without posterior crossbite were 2.2 times more likely to have bruxism in comparison to children with posterior crossbite. Children who complained of having headaches were 1.5 times more likely (50% more) to develop bruxism, in comparison to those who did not have headaches. Children with restless sleep have 2.1 more chances to develop bruxism, in comparison to children with peaceful sleep.

**Table 7:** Results of logistic regression model applied for the presence of bruxism.

Characteristic	Chance ratio	95% confidence interval		
		Lower limit	Upper limit	P-value
**Posterior crossbite**				
Present	1.0	1.4	3.6	0.0011
Absent	2.2			
**Headache**				
No	1.0	1.1	2.2	0.0148
Yes	1.5			
**Restless sleep**				
No	1.0	1.6	2.9	‹0.0001
Yes	2.1			

## DISCUSSION

The prevalence of the parafunctional habit of bruxism in deciduous dentition has been the subject of some recent epidemiological studies. However, lack of uniformity and standardization of criteria to assess bruxism in children have resulted in a wide variation of its prevalence.^17^^,^ 22 ^,^ 25 ^,^ 28

The actual occurrence of bruxism in children cannot be easily registered because the presence of wear facets observed during clinical examination may indicate only a history of bruxism which may no longer be occurring at the time of evaluation. On the other hand, the beginning of the habit may not yet have caused the tooth to show signs of wear. Thus, although being subjective, the method of interviewing the children's parents is considered trustworthy to assess the prevalence of this habit, as it reflects the existence of dental noises produced by the child and which are actually perceived by parents. Therefore, even though prevalence was underestimated, the occurrence of false-positives is almost eliminated.^29^

The methods employed herein included a questionnaire answered by parents and/or guardians and followed a similar standard used in other studies.^5^^,^ 13 ^,^ 14 ^,^ 17 ^,^ 30 The difficulty in diagnosing this parafunctional disturbance is widely known^29^ even when diagnosis is based on polysomnography exams which were not performed in this sample. Such an exam becomes even more limited when dealing with children at the stage of primary dentition.

Results obtained herein showed a high prevalence of the parafunctional habit of bruxism, thus corroborating results of previous studies.^11^^,^ 16 ^,^ 17 ^,^ 18 ^,^ 24 ^,^ 30 However, some studies^21^^,^ 27 revealed even higher occurrences of around 45% whereas others revealed much lower occurrences as low as 6.4% in Turkey^1^ and 11% in India.^5^ It can be supposed that the origin of these differences (regarding the prevalence of bruxism in children) is possibly related to lack of uniformity in methodological research, cultural differences between countries, or even a variety of age groups.

Regarding patients' sex, some studies show that the parafunctional habit of bruxism in children has prevailed for males,^14^^,^ 17 ^,^ 18 which was also found in this study. However, a study of Mexican children detected the presence of bruxism in 6% in female population compared to 1.5% in male population.^12^ Other studies found no significant differences regarding sexual dimorphism.^5^^,^ 7 ^,^ 9 ^,^ 28

In the present study, the majority of children with bruxism did not present posterior crossbite when assessed statistically. However, the results support statistical analysis, thereby giving significance to factors of bruxism, headache and restless sleep. Bruxism is often associated with patient's emotional state and is more common among anxious children.^6^^,^ 23

When the chi-square test was applied, the only factors showing a statistically significant association were headache and restless sleep. Nevertheless, posterior crossbite was not significantly associated with headache and restless sleep. These data confirm what the literature already proves: There is a correlation between the presence of restless sleep in children with headache.^8^^,^ 10

The logistic regression model showed that children without posterior crossbite were 2.2 more likely to have bruxism, in comparison to children with posterior crossbite. Children who complained of headache were 1.5 times more likely to develop bruxism, in comparison to those who did not complain of headaches. Also, children with restless sleep were 2.1 more likely to have bruxism, in comparison to children with peaceful sleep.

Researchers believe bruxism is strongly correlated with children's emotional state^6^^,^ 8 ^,^ 10 ^,^ 17 ^,^ 23 (anxiety, hyperactivity, restless sleep), which was also considered in this paper. Additionally, according to some studies,^4^^,^ 14 ^,^ 18 ^,^ 20 ^,^ 21 ^,^ 28 children's general health, occurrence of malocclusion (absent, mild, moderate and severe) and the presence of other parafunctional oral habits can also be paired with bruxism. However, in contrast to these studies, the results of the present study showed no significant relationship between the presence of bruxism and posterior crossbite, i.e., the occlusal factor.

In this context, the combination of several etiologic factors present in a single individual greatly increases the likelihood of bruxism in children. Furthermore, its high prevalence is being considered an intrinsic problem of modern society. Thus, different treatment modalities available nowadays should be individualized for each patient.

Early diagnosis is important to reduce the consequences of this parafunctional activity. Clinician's treatment protocol should be directed towards reducing psychological stress and treating signs and symptoms, since bruxism cannot be permanently eliminated. One should opt for conservative therapy, with reversible and not invasive control as well as continuous follow-up. Due to its multifactorial etiology, bruxism treatment should involve professionals such as pediatricians, psychologists, pediatric dentists and otolaryngologists.

## CONCLUSIONS

The parafunctional habit of bruxism was prevalent in 28.8% of the total sample studied. There was no significant relationship between this parafunctional habit and the transverse plane of occlusion. However, headache and restless sleep were significantly associated with bruxism in children.
